# Cigarette smoke promotes IL-6-dependent lung cancer migration and osteolytic bone metastasis

**DOI:** 10.7150/ijbs.94339

**Published:** 2024-06-03

**Authors:** Jeng-Hung Guo, Le Huynh Hoai Thuong, Ya-Jing Jiang, Chang-Lun Huang, Yu-Wen Huang, Fang-Ju Cheng, Po-I Liu, Chun-Lin Liu, Wei-Chien Huang, Chih-Hsin Tang

**Affiliations:** 1 Graduate Institute of Biomedical Sciences, China Medical University, Taichung, Taiwan.; 2 Department of Neurosurgery, China Medical University Hospital, Taichung, Taiwan.; 3 Division of General Thoracic Surgery, Department of Surgery, Changhua Christian Hospital, Changhua, Taiwan.; 4 Department of Physical Therapy, Asia University, Taichung, Taiwan.; 5 Department of General Thoracic Surgery, Asia University Hospital, Taichung, Taiwan.; 6 Center for Molecular Medicine, China Medical University Hospital, Taichung, Taiwan.; 7 Department of Pharmacology, School of Medicine, China Medical University, Taichung, Taiwan.; 8 Department of Medical Laboratory Science and Biotechnology, College of Medical and Health Science, Asia University, Taichung, Taiwan.; 9 Chinese Medicine Research Center, China Medical University, Taichung, Taiwan.; 10 Department of Medical Research, China Medical University Hsinchu Hospital, Hsinchu, Taiwan.

**Keywords:** Cigarette smoke, Lung cancer, IL-6, Bone metastasis, Osteoclast

## Abstract

Lung cancer stands as a major contributor to cancer-related fatalities globally, with cigarette smoke playing a pivotal role in its development and metastasis. Cigarette smoke is also recognized as a risk factor for bone loss disorders like osteoporosis. However, the association between cigarette smoke and another bone loss disorder, lung cancer osteolytic bone metastasis, remains largely uncertain. Our Gene Set Enrichment Analysis (GSEA) indicated that smokers among lung cancer patients exhibited higher expression levels of bone turnover gene sets. Both The Cancer Genome Atlas (TCGA) database and our clinic samples demonstrated elevated expression of the osteolytic factor IL-6 in ever-smokers with bone metastasis among lung cancer patients. Our cellular experiments revealed that benzo[α]pyrene (B[α]P) and cigarette smoke extract (CSE) promoted IL-6 production and cell migration in lung cancer. Activation of the PI3K, Akt, and NF-κB signaling pathways was involved in cigarette smoke-augmented IL-6-dependent migration. Additionally, cigarette smoke lung cancer-secreted IL-6 promoted osteoclast formation. Importantly, blocking IL-6 abolished cigarette smoke-facilitated lung cancer osteolytic bone metastasis *in vivo*. Our findings provide evidence that cigarette smoke is a risk factor for osteolytic bone metastasis. Thus, inhibiting IL-6 may be a valuable therapeutic strategy for managing osteolytic bone metastasis in lung cancer patients who smoke.

## Introduction

Annually, around 2 million cases and 1.8 million fatalities of lung cancer are reported, resulting it the most general cause of cancer-related mortality worldwide 1, 2. Various risk factors, including smoking, radiation, air pollution, and heredity, participate to the development of lung cancer 3, 4. Cigarette smoke contains over 7,000 chemical components, such as nicotine, nitrosamines, and benzene, making it the primary risk factor for lung cancer 5. Compared to non-smokers, lifetime smokers face an average 20-fold higher risk of developing lung cancer 1, 6. Crucially, individuals with lung cancer who continue to smoke experience lower overall survival and quality of life, increased likelihood of recurrence, and reduced efficacy from therapies such as chemotherapy 7.

Metastasis is the term used to describe the development of secondary tumors that migrate through the lymphatic or circulatory systems and form farther away from the original cancer location 8. Metastasis is responsible for around 90% of cancer-related fatalities 9. Between 30% and 40% of patients with advanced lung cancer will progress to bone metastases; the majority of these are osteolytic, which can lead to pathological fractures and poor clinical outcomes 10, 11. Interleukin (IL) and receptor activator of nuclear factor-kappa B ligand (RANKL), two osteolytic proteins secreted by metastatic bone cancer cells, promote osteoclast bone resorption and facilitate the secretion of immobilized growth factors from the bone matrix 12. Interaction between osteoclasts and cancer cells induces an aggressive tumor phenotype with the potential for metastatic dissemination and bone degradation 13. Treatments for bone metastases, such as denosumab and bisphosphonates, are based on interrupting osteoclastic resorption 14, 15. Although these medications significantly decrease the quantity of osteoclasts and the degree of bone resorption, their side effects are extremely concerning 16.

Cigarette smoking is a major risk factor for lung cancer and plays a key role in lung cancer progression, metastasis and survival 17. While cigarette smoking has been confirmed to be deleterious to bone remodeling by adversely affecting osteoclast and osteoblast functions 18, 19, the association between cigarette smoking and lung cancer with bone metastasis has never been thoroughly discussed. Our investigation of records from the Gene Expression Omnibus (GEO) database exhibited upregulated bone remodeling, bone resorption and bone formation genes in lung cancer patients who were cigarette smokers compared with nonsmokers. In both *in vitro* and *in vivo* data, we demonstrated that long-term exposure to cigarette smoke extract (CSE) and its carcinogen polycyclic aromatic hydrocarbon benzo[α]pyrene (B[α]P) elevates IL-6 generation from lung cancer cells through the PI3K, Akt, and NF-κB signaling pathway, subsequently enhancing lung cancer migration and metastasis. We noted that high levels of IL-6 in lung cancer cells exposed to CSE and B[α]P stimulated IL-6, promoting osteoclastogenesis. Our results emphasize the value of further examinations into strategies that block IL-6 to abolish lung cancer migration and osteolytic bone metastasis in smokers with lung cancer.

## Materials and methods

### Materials

IL-6 antibody was purchased form R&D Systems (MA, USA). p85, Akt and p65 antibodies were purchased from Santa Cruz Biotechnology (CA, USA). Antibodies against the phosphorylated forms of p85, Akt and p65 as well as integrin β3, MMP9, CTSK and DC-STAMP were obtained from Cell Signaling Technology (Danvers, MA, USA). All ON-TARGETplus siRNAs were sourced from Dharmacon (Lafayette, CO, USA). All other chemical reagents not already mentioned were obtained from Sigma-Aldrich (St. Louis, MO, USA).

### Cell culture

Human A549 cells line was purchased from American Type Culture Collection (Manassas, VA, USA); the murine RAW 264.7 cell line was obtained from the Bioresource Collection and Research Center (Hsinchu, Taiwan). Human A549 cells were exposed to 1% CSE or 1 μΜ B[α]P for 30 weeks (A549^CSE^ and A549^B[α]P^) was generated according our previous publications 20, 21. The A549^CSE^-Luc and A549^B[α]P^-Luc luciferase-expressing cell lines, which contain the firefly luciferase gene, were established according to our previous report 20. The luciferase activities were detected using a luciferase assay (Supplementary Fig. S1). To clone IL-6 knockdown A549^CSE^-Luc and A549^B[α]P^-Luc cells, a lentiviral vector capable of expressing IL-6-specific shRNA was obtained from the National RNAi Core (Taipei, Taiwan). Cells were seeded in a 6-well dish and the lentivirus was applied to the medium (multiplicity of infection = 10). After 24 h, the culture medium was changed and then at 48 h, 2 μg/mL of puromycin was applied to select for IL-6 shRNA-expressing cells. Cells were cultured in RPMI 1640 medium (Gibco, USA) supplemented with 10% fetal bovine serum (FBS; Gibco, Grand Island, NY, USA), penicillin-streptomycin (Gibco, Grand Island, NY, USA), and incubated at 37°C in a humidified environment containing 5% CO_2_.

### Analyses of databases

GEO dataset (GSE31210) involving 123 ever-smokers and 123 never-smokers was analyzed for IL-6 gene expression in the lung cancer tissues. Gene set enrichment analysis (GSEA) software was analyzed the bone remodeling gene expression when lung cancer cells are subjected to cigarette smoke. Associations between levels of IL-6 and RANKL protein expression and survival prognoses of lung cancers smoker were examined in records downloaded from the Kaplan-Meier database. Records from The Cancer Genome Atlas (TCGA) database were analyzed for levels of IL-6 expression in never-smokers and ever-smokers in lung cancer patients.

### Ingenuity Pathway Analysis (IPA)

A list of differentially expressed human lung cancer patient genes from the GEO database (GSE31210), including gene names and associated expression levels, was imported into the IPA program (Qiagen). The differentially expressed data, comprising biological processes, signaling pathways, regulation transcriptional factors, and gene networks, were analyzed using the software's "core analysis" tool 22.

### Western blot

Cells were subjected to lysis using 100 μl of RIPA lysis buffer supplemented with a protease inhibitor cocktail (Roche, Indianapolis, IN, USA). The subsequent retrieval of supernatants was carried out as part of a western blot analysis, with comprehensive methods outlined in our prior research publications 23, 24.

### mRNA expression analysis

Total RNA was isolated from lung cancer cells using TRIzol reagent (MDBio Inc., Taipei, Taiwan). Subsequently, we conducted reverse transcription of 2 μg of total RNA to generate complementary DNA (cDNA) using the M-MLV RT kit, following the respective manufacturer's protocols. For qPCR assays, we employed the StepOnePlus™ Real-Time PCR System (Applied Biosystems) 25-27.

### Enzyme-linked immunosorbent assay (ELISA)

In order to analysis the production of IL-6, we quantified their expression levels in cell culture medium using ELISA kits (R&D Systems, MA, USA) designed for IL-6 24, 28, 29.

### Migration assay

Cell migration assays were performed using Track Etched Membrane (8 μm pore size; GVS North America Sanford, ME, USA). Lung cancer cells (1 × 10^4^) were applied with pharmacological inhibitors or genetic siRNA then seeded into the upper chamber. After 18 h, migratory cells were stained with crystal violet and manually counted under a microscope.

### Osteoclast differentiation

RAW 264.7 cells (2000 cells) were cultured incubated with lung cancer condition medium (CM). Tartrate-resistant acid phosphatase (TRAP)-positive multinucleated (N3 nuclei) cells that were identified by the TRAP kit were classified as mature osteoclasts after 7 days 30.

### Luciferase activity

Lung cancer cells were transfected with NF-κB luciferase plasmid (Stratagene; MO, USA) using Lipofectamine 2000 (Invitrogen; Carlsbad, CA, USA) then applied with pharmacological inhibitors or genetic siRNA for 24 hrs. Luciferase activity was calculated using the dual luciferase assay system according the manufacturer's procedures 31.

### Osteolytic bone metastasis animal model

Lung cancers (1 × 10^6^) were injected into the caudal artery in the tail of six-week-old male nude mice purchased from BioLASCO Taiwan Co., Ltd. (Taipei, Taiwan). After 8 weeks, the IVIS® Spectrum imaging system was used to determine the *in vivo* tumor mass. All mice were humanely sacrificed and tumor-induced bone erosion was evaluated by X-ray. The specimens counterstained with H&E and observed under a light microscope for histological changes. The animal study was approved by the Institutional Animal Care and Use Committee of China Medical University (Approval No.: CMUIACUC-2022-237).

### Immunohistochemistry (IHC) staining

The tumor specimens from lung cancer patients were granted approval by the Ethics Review Board of Asia University Hospital (No. CMUH111-REC3-094). Patients provided informed written consent. The sections underwent deparaffinization with xylene followed by rehydration using ethanol. Subsequently, they were subjected to overnight incubation at 4°C with specific primary antibodies, following a previously established protocols 27, 32, 33. Two independent observers, who were blinded to the histopathological data, scored the IHC staining intensity.

### Statistical analysis

All quantified outcomes are based on a minimum of three experiments and are expressed as the mean value along with the standard deviation (SD). Statistical analyses were carried out using GraphPad Prism 8.0 (GraphPad Software, La Jolla, CA, USA). Significance was determined when the *p*-value was less than 0.05.

## Results

### The expression of IL-6 is associated with bone metastases from lung cancer in smokers

**Figure 1:**
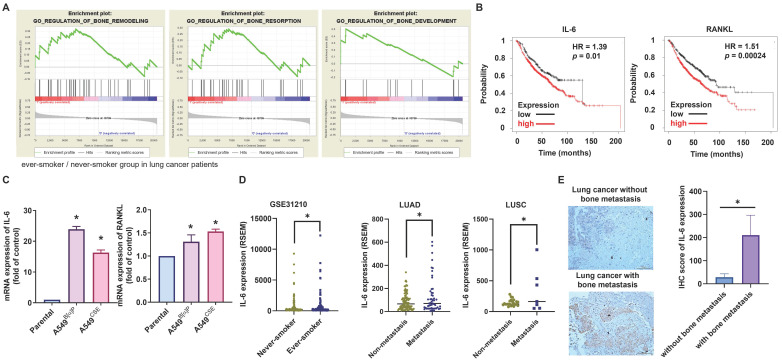
** Association of IL-6 levels with bone metastases from lung cancer in smokers.** (A) GSEA-based pathway analysis included bone remodeling, bone resorption, and bone formation gene sets linked with lung cancer in smokers and nonsmokers obtained from the BioCarta database. (B) Kaplan-Meier analysis determined levels of IL-6 (low expression n=215, high expression n=605) and RANKL (low expression n=310, high expression n=510) and overall survival rates of patients with lung cancer. (C) The IL-6 mRNA expression in indicated lung cancer cells was examined by qPCR. (D) The GEO and TCGA database revealed IL-6 expression in never-smoker and ever-smoker lung cancer samples, as well as in LUAD and LUSC tissue samples. (E) IHC staining of IL-6 in lung cancer patients with or without bone metastasis (n=7 in each group). *p < 0.05 versus the parental group.

Cigarette smoking, known to cause aberrant regulation of different signaling transduction mechanisms, is associated with genetic abnormalities and tumor advancement 34. Little information currently exists on the relationship between cigarette smoking in lung cancer patients and bone turnover and bone metastasis. Applying GSEA to the BioCarta records in the GEO database exhibited that smokers among lung cancer patients had higher expression levels of three bone turnover gene sets, including bone remodeling, bone resorption, and bone formation (Fig. 1A). IL-6 and RANKL, two essential components of bone resorption and metastasis 12, showed increased expression levels in ever-smokers according to Kaplan-Meier analysis, correlating with proportionately reduced overall survival probabilities (Fig. 1B). In our established long-term exposure model to 1% CSE or 1μM B[α]P, IL-6 and RANKL mRNA and protein expression significantly increased in A549^B[α]P^ and A549^CSE^ cells compared with parental cells, with IL-6 showing greater upregulation than RANKL (Fig. 1C; Supplementary Fig. S2). Analysis of TCGA database results revealed elevated expression of IL-6 in ever-smokers with metastasis in lung adenocarcinoma (LUAD) and lung squamous carcinoma (LUSC) patients (Fig. 1D). Our clinical samples also confirmed higher IL-6 expression in lung cancer patients with bone metastasis compared to those without bone metastasis (Fig. 1E). Thus, IL-6 levels are linked with lung cancer bone metastasis in smoker.

### The PI3K, Akt and NF-κB signaling pathways are mediated in cigarette smoke-enhanced IL-6-dependet cell migration

**Figure 2:**
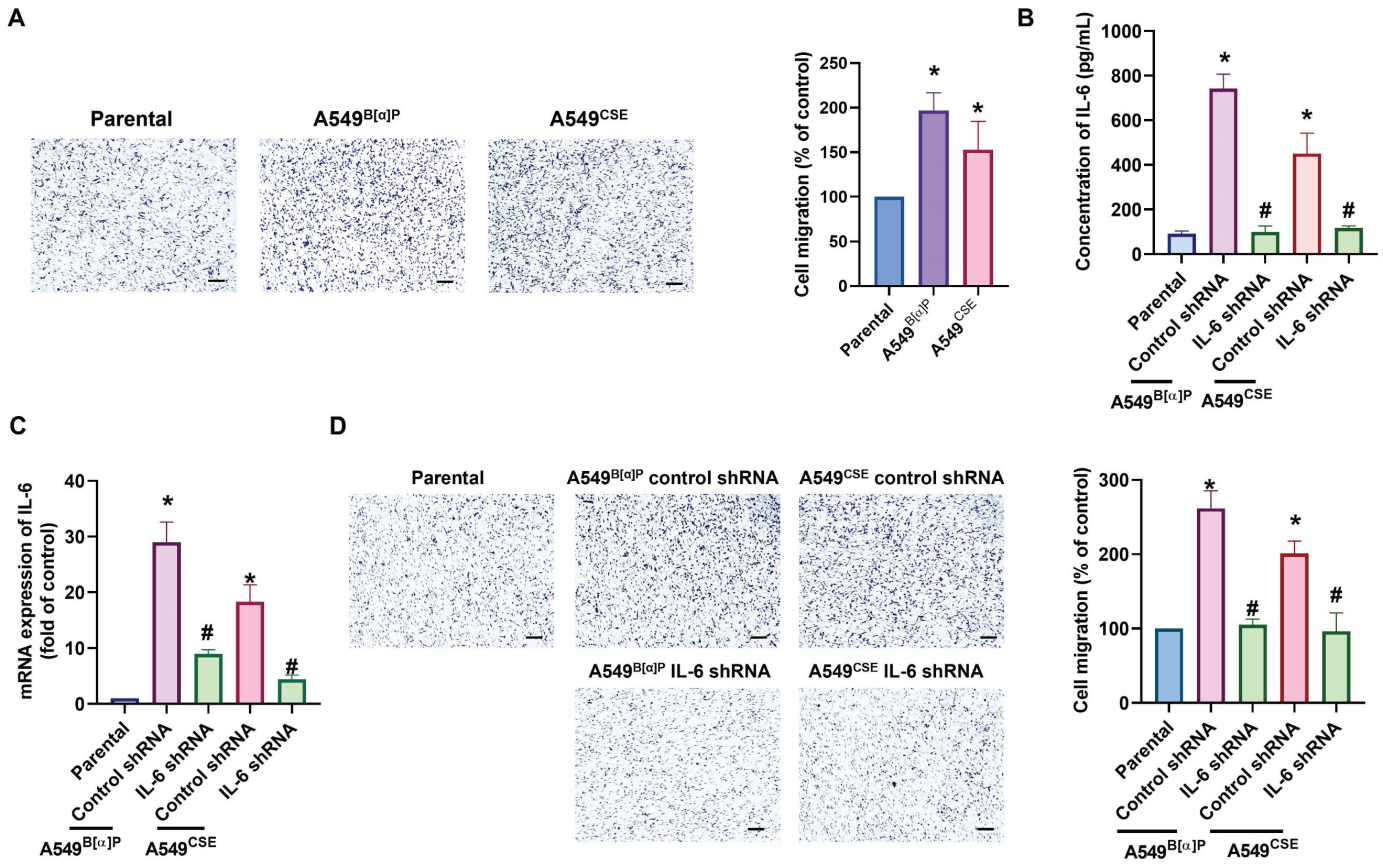
** Cigarette smoke promotes IL-6-dependent lung cancer migration.** (A) The cell migration ability in indicated cells was examined. (B-D) Lung cancer cells were transfected with IL-6 shRNA, and IL-6 expression and cell migration were examined by ELISA, qPCR and migration assay. *p < 0.05 versus the parental group; #*p* < 0.05 versus the control-shRNA group.

**Figure 3:**
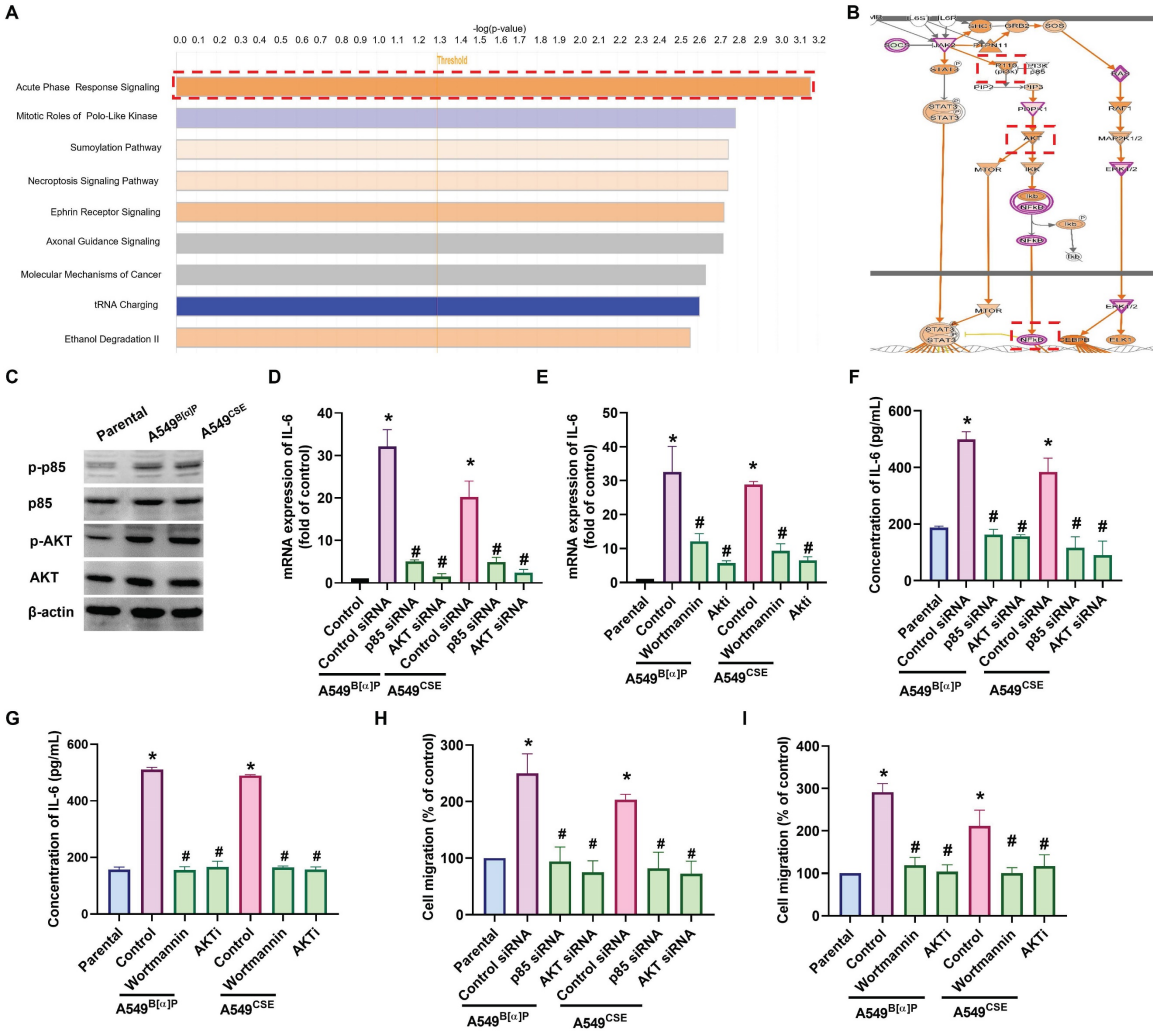
** PI3K and Akt pathways regulate cigarette smoke-induced IL-6 expression and cell migration in lung cancer.** (A&B) IPA pathway enrichment figure showing pathways in the GSE31210 database that were markedly elevated. (C) The phosphorylation of p85 and Akt in indicated cells was examined by western blot. (D-I) Cells were pretreated with wortmannin and Akti or transfected with p85 and Akt siRNA, and IL-6 expression and cell migration were examined by ELISA, qPCR and migration assay. *p < 0.05 versus the parental group; #*p* < 0.05 versus the control-siRNA group.

Next, we directly examined the functions of cigarette smoke on lung cancer motility. The A549^B[α]P^ and A549^CSE^ cells exhibited significantly higher migration abilities than parental cells and correlated with IL-6 concentration (Fig. 2A). Transfection of A549^B[α]P^ and A549^CSE^ cells with IL-6 shRNA reduced IL-6 production and cell migration (Fig. 2B-D). These results indicate that cigarette smoke promotes IL-6-dependent cell migration in human lung cancer. To investigate the mechanisms underlying cigarette smoke in lung cancer by analyzing molecular cascades in the GSE31210 database records using IPA, we found that the PI3K, Akt, and NF-κB mechanisms were related to the top signaling cascade, namely, the acute phase response signaling 35 (Fig. 3A&B). In our experiments, A549^B[α]P^ and A549^CSE^ cells enhanced the phosphorylation of p85 and Akt (Fig. 3C). Applying A549^B[α]P^ and A549^CSE^ cells with PI3K inhibitor (wortmannin) and an Akt inhibitor (Akti), or transfecting them with p85 and Akt siRNAs, blocked cigarette smoke-facilitated induction in IL-6 expression and cell migration (Fig. 3D-I; Supplementary Fig. S3).

**Figure 4:**
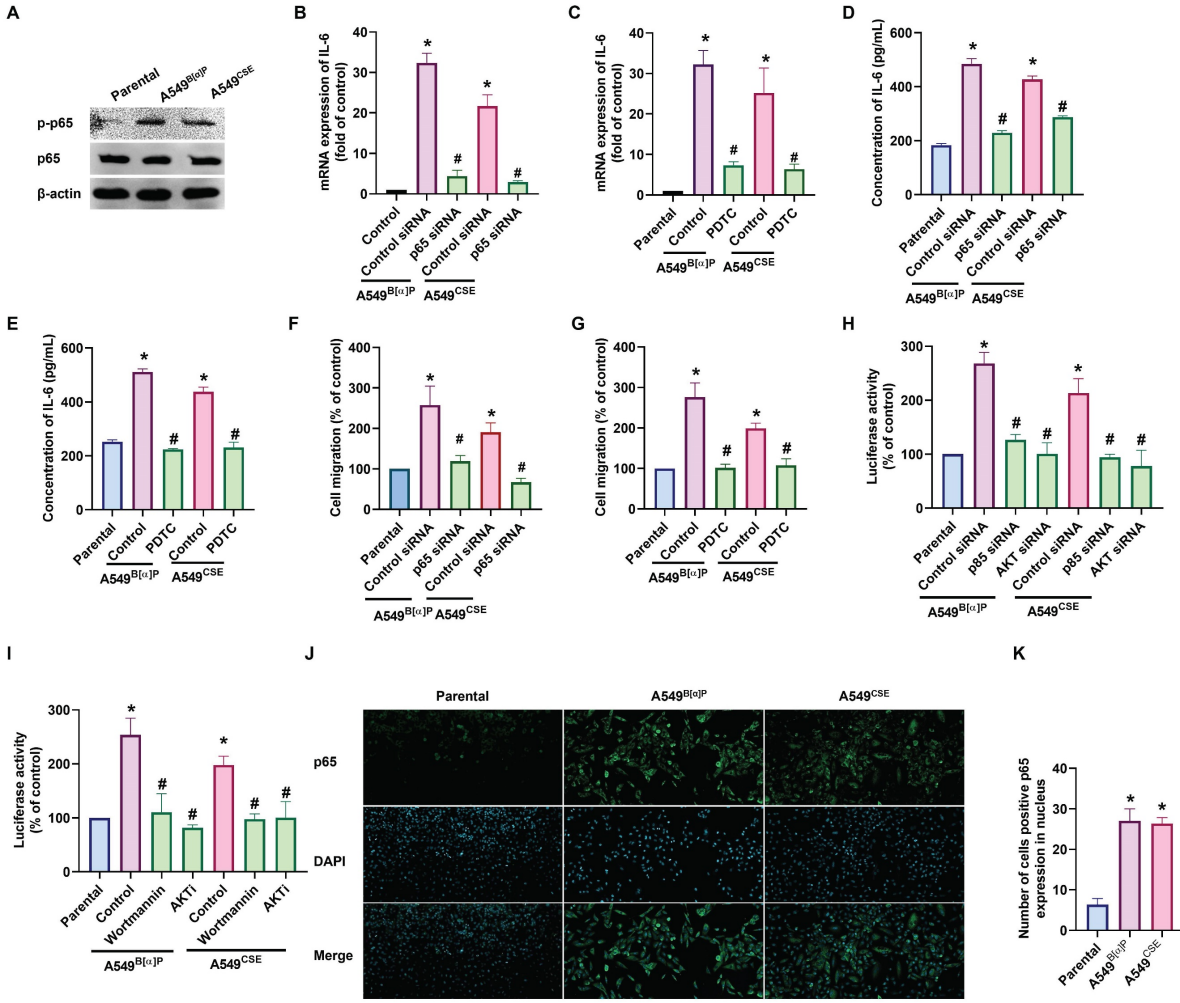
** NF-κB transactivation is involved in cigarette smoke-promoted IL-6-dependent cell migration in lung cancer.** (A) The phosphorylation of p65 in indicated cells was examined by western blot. (B-G) Cells were pretreated with PDTC or transfected with p65 siRNA, and IL-6 expression and cell migration were examined. (H&I) Cells were pretreated with wortmannin and Akti or transfected with p85 and Akt siRNA, and NF-κB luciferase activity was examined. (J&K) The immunofluorescence staining of p65 in indicated cells was examined. *p < 0.05 versus the parental group; #*p* < 0.05 versus the control-siRNA group.

We then investigated whether NF-κB regulates cigarette smoke-facilitated cell migration in lung cancer. We observed that the A549^B[α]P^ and A549^CSE^ cells exhibited up-regulated phosphorylation of p65 and translocation of p65 into the nucleus (Fig. 4A&J&K). Treatment of cells with the NF-κB inhibitor (PDTC) abolished cigarette smoke-enhanced IL-6 synthesis and cell migration (Fig. 4B-G; Supplementary Fig. S4). Additionally, transfection with p65 siRNA produced similar effects (Fig. 4B-G; Supplementary Fig. S4). Furthermore, A549^B[α]P^ and A549^CSE^ cells enhanced the luciferase activity of NF-κB (Fig. 4H&I). Moreover, both PI3K and Akt inhibitors antagonized cigarette smoke-promoted NF-κB luciferase activity (Fig. 4H&I). IL-6 promoter constructs containing mutation at the NF-κB site were used to confirm whether NF-κB directly influenced the transcriptional activity of IL-6. Indeed, the enhanced IL-6 promoter activity in A549^B[α]P^ and A549^CSE^ cells was abolished by mutation in the NF-κB binding site (Supplementary Fig. S5), indicating that NF-κB transcriptional activity is crucial in cigarette smoke-induced IL-6 synthesis and cell migration through the PI3K and Akt pathways.

### Cigarette smoke lung cancer-secreted IL-6 promotes osteoclastogenesis

**Figure 5:**
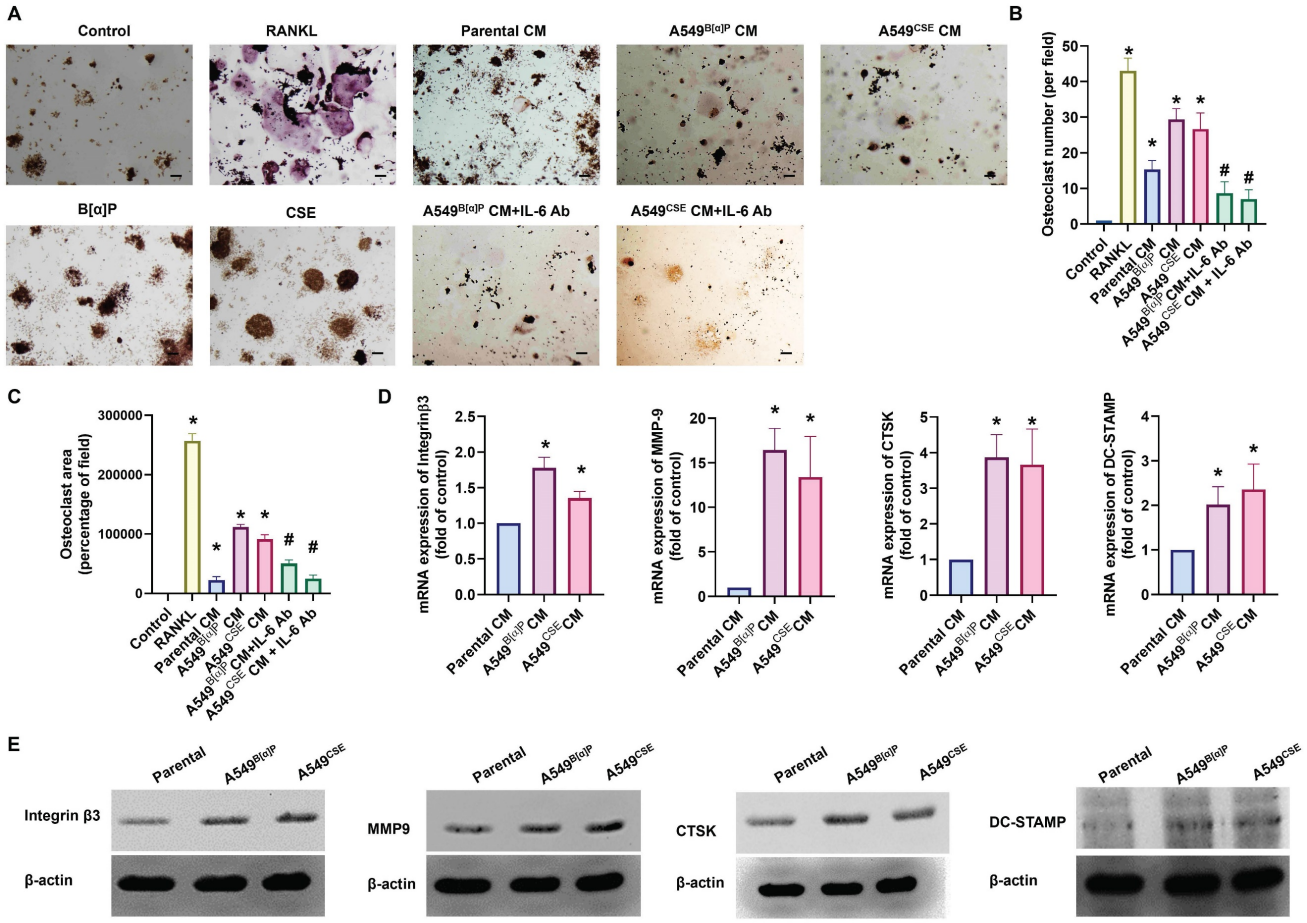
** Cigarette smoke lung cancer-secreted IL-6 promotes osteoclast formation.** (A-C) RAW 264.7 cells were treated with lung cancer CM with or without IL-6 antibody (1 µg/ml) for 7 days, the mature osteoclast was determined by TRAP staining. (D&E) qPCR and Western blot analysis results showing integrin β3, MMP9, CTSK, and DC-STAMP expression in RAW 264.7 cells treated with lung cancer CM for 7 days. *p < 0.05 versus the parental group; #*p* < 0.05 versus the A549^B[α]P^ or A549^CSE^ CM group.

When lung cancer spreads to the bone, it often leads to pain and fractures due to bone destruction 36. We investigated whether cigarette smoke exacerbates bone erosion caused by lung cancer. Compared with parental cells, A549^B[α]P^ and A549^CSE^ cell-conditioned media (CM) markedly induced osteoclast formation from RAW264.7 cells, as assessed by counting TRAP-positive cells and measuring the osteoclast area (RANKL-induced osteoclast formation was used as a positive control) (Fig. 5A-C). qPCR and Western blot analysis of osteoclast markers, including integrin β3, MMP9, CTSK, and DC-STAMP, also revealed a similar phenomenon (Fig. 5D&E). Interestingly, treatment with an IL-6 antibody (1 µg/ml) in CM significantly abolished A549^B[α]P^ and A549^CSE^ cells-CM-induced promotion of osteoclastogenesis (Fig. 5A-C). The osteoclastogenesis from primary human monocytes has similar results (Supplementary Fig. S6). Therefore, IL-6 plays a key role in cigarette smoke lung cancer-induced osteoclast formation.

### Blocking IL-6 abolishes cigarette smoke-induced lung cancer osteolytic bone metastasis

**Figure 6:**
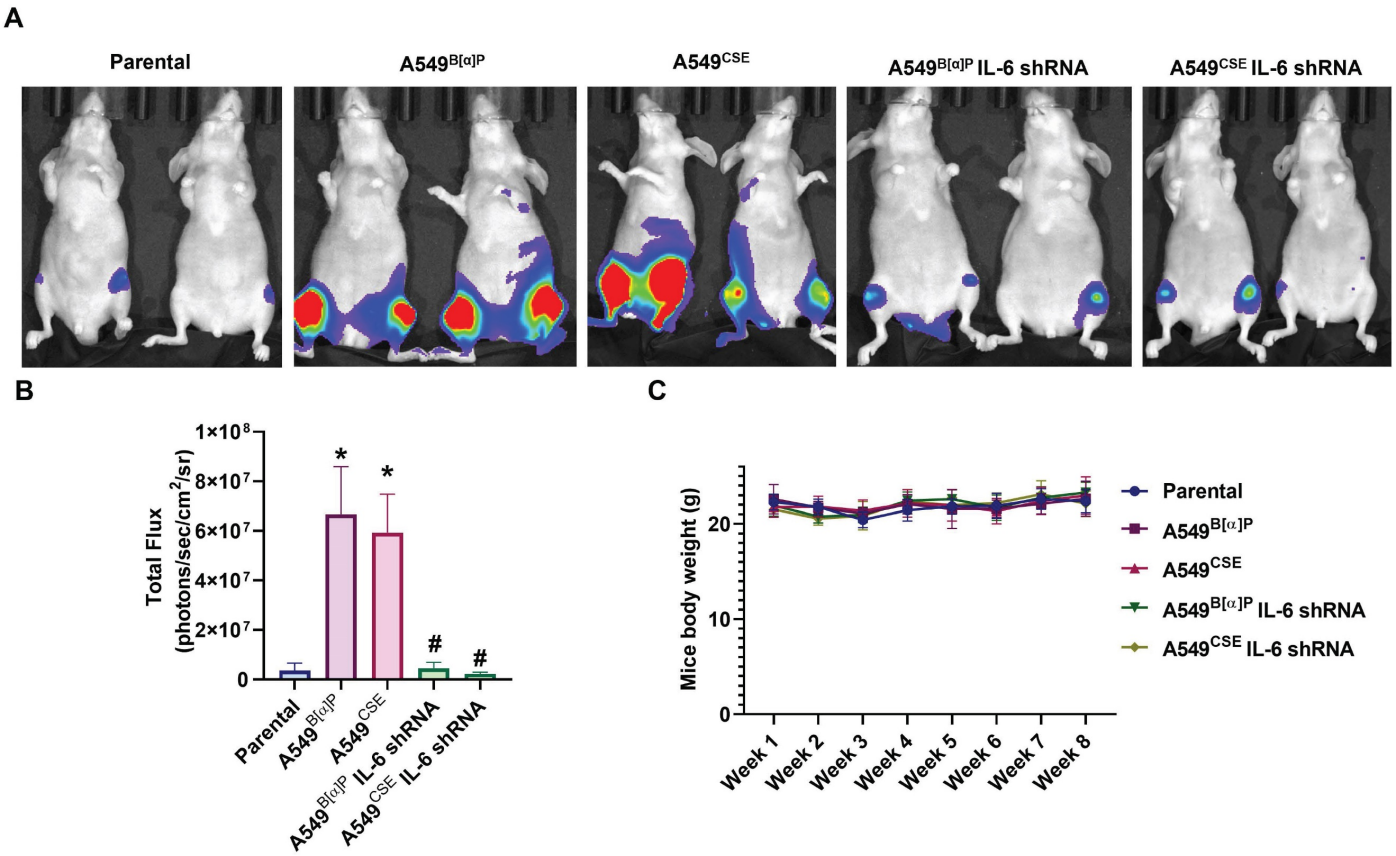
** Blocking IL-6 inhibits cigarette smoke-induced lung cancer metastasis to bone *in vivo*.** (A&B) Representative bioluminescent images of bone metastasis at 8 weeks after caudal artery injections of indicated lung cancer cell lines into nude mice. (C) Mouse body weights were measured weekly after caudal artery. *p < 0.05 versus the parental group; #*p* < 0.05 versus the A549^B[α]P^ or A549^CSE^ group.

**Figure 7:**
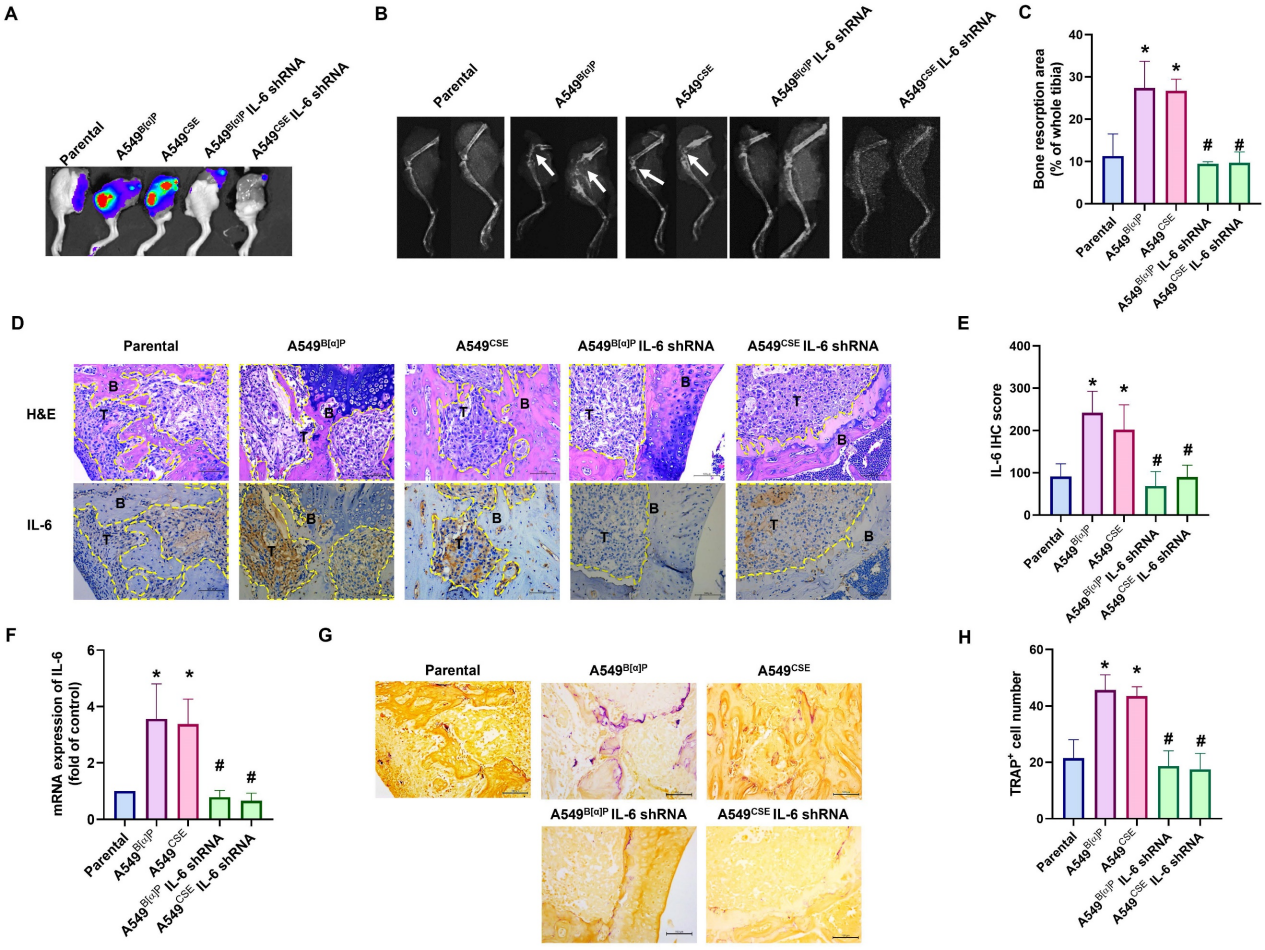
** Blocking IL-6 abolishes cigarette smoke-promoted lung cancer osteolytic bone metastasis.** (A-C) Representative bioluminescent and X-ray images of bone erosion in each study group. (D&E) Representative H&E and immunohistochemical images of IL-6 in bone specimens from mice. (F) The IL-6 mRNA expression in blood from mice was examined by qPCR using human IL-6 primers. (G&H) Representative TRAP-positive staining images of mouse leg bones. *p < 0.05 versus the parental group; #*p* < 0.05 versus the A549^B[α]P^ or A549^CSE^ group.

Next, we injected lung cancer cells into the caudal arteries of nude mice to investigate lung cancer osteolytic bone metastasis *in vivo*. After an 8-week injection, there were no variations in body weights between the groups (Fig. 6C). However, compared with parental cells, A549^B[α]P^ and A549^CSE^ cells significantly metastasized to bone (Fig. 6A&B). Conversely, knockdown of IL-6 reduced B[α]P and CSE-facilitated metastasis to bone, as observed through *in vivo* bioluminescence imaging (Fig. 6A&B). X-ray findings exhibited inhibited tumor growth and bone erosion in mice bearing knockdown IL-6 tumors (Fig. 7A-C). H&E staining demonstrated the osteolytic nature of the bone lesions formed by A549^B[α]P^ and A549^CSE^ cells and their inhibition by stable knockdown of IL-6 cells (Fig. 7D). IHC staining of tumor tissue and qPCR of blood mice levels of IL-6 were markedly inhibited in stable knockdown IL-6 cells (Fig. 7D-F). The loss of IL-6 expression was linked to significantly lower levels of TRAP-positive osteoclasts (Fig. 7G&H), consistent with our findings that cigarette smoke regulates osteoclast development through IL-6-dependent pathways.

## Discussion

Compared to nonsmokers, patients with lung cancer who smoke experience worse survival rates and more tumor recurrences 27. Cigarette smoke influences the growth, migration, invasion, and medication resistance of lung cancer 28. Attenuation of bone homeostasis, enhanced bone resorption, and decreased bone formation are associated with metastatic bone disease 45. However, the relationship between cigarette smoke and osteolytic bone metastasis in lung cancer is still unknown. Our results suggest that cigarette smoke enhances the PI3K/Akt-activating NF-κB signaling pathways to facilitate IL-6 synthesis and cell motility in lung cancer cells. The IL-6 secretion from metastatic lung cancer promotes osteoclast formation, resulting in osteolytic bone metastasis. We also found similar results in another lung cancer cell line, H292. The H292^B[α]P^ and H292^CSE^ cells exhibited higher IL-6 level and migration abilities compared to the parental cells (Supplementary Fig. S7). Transfection of IL-6, p85, Akt and p65 siRNA all blocked cigarette smoke-enhanced IL-6 synthesis and cell migration (Supplementary Fig. S8). In addition, an IL-6 antibody abolished H292^B[α]P^ and H292^CSE^ cells-CM-induced promotion of osteoclastogenesis (Supplementary Fig. S9). Our findings suggest that targeting IL-6 may be a worthwhile therapeutic strategy in the management of lung cancer in smokers.

There are around 7,000 different compounds included in cigarette smoke 37. Cigarette smoke mixtures were shown to include over 60 carcinogens, such as B[α]P, N'-nitrosonornicotine (NNN), benzene, and catechol, which were identified in the mixtures of cigarette smoke 38. Our previous report indicated that B[α]P but not NNN, among the CSE-derived carcinogens, renders lung cancer cells more resistant to EGFR tyrosine kinase inhibitors 21. According to the International Organization for Standardization (ISO), the average B[α]P content is 0.92-3.46 ng per cigarette 39. In the current study, we found that both CSE and B[α]P displayed the same effects on IL-6 production, activating the PI3K/Akt/NF-κB pathway, cell motility, osteoclast formation, as well as osteolytic bone metastasis *in vivo*. We, therefore, suggest that B[α]P is the major substance in CSE that controls IL-6-dependent bone metastasis. However, whether other substances are also involved in CSE's effects needs further investigation.

Bone loss is a significant concern for patients with osteoporosis and osteolytic bone metastasis. Anti-resorption drugs, such as bisphosphonates and denosumab, are used to treat bone loss 40, 41. Cigarette smoking, one of the most prevalent harmful habits and the world's greatest avoidable cause of death, has been established as a separate risk factor for the progression of osteoporosis 42, 43. The human body absorbs and retains cigarette smoke, disrupting the process of dynamic equilibrium. This disruption leads to a reduction in osteogenesis in osteoblasts and an increase in resorption in osteoclasts, causing osteoporosis—a condition marked by a reduce in bone mass and deterioration in bone structure 44. However, whether cigarette smoke is associated with osteolytic bone metastasis remains largely uncertain. Our GSEA analysis indicated that smokers among lung cancer patients had higher expression levels of three bone turnover gene sets including bone remodeling, bone resorption, and bone formation. Our cellular and preclinical models determined that B[α]P and CSE facilitate lung cancer migration and osteolytic bone metastasis. To the best of our knowledge, this investigation is the first to provide experimental data that cigarette smoke is a risk factor for osteolytic bone metastasis.

Originally discovered as a cytokine generated from T cells, IL-6 has a wide range of biological functions in different tissues and cell types 45. Numerous researchers have documented that IL-6 can promote the growth of cancer cells 46, 47. It has also been demonstrated that IL-6 is present in or synthesized by a range of malignant tumors, including lung cancer, and autocrine growth activation has been proposed as a potential mechanism of action 48, 49. Moreover, IL-6 affects bone cells in a special and significant way, promoting the development of cells that resemble osteoclasts and are capable of resorbing bone 50. Additionally, it has been documented that blocking osteoclastic bone resorption with a neutralizing antibody against human IL-6 restored hypercalcemia linked to cancer cells 51. Here, we demonstrated two important roles of IL-6 in cigarette smoke-promoted lung cancer osteolytic bone metastasis. First, IL-6 controls cigarette smoke-induced lung cancer migration, as demonstrated with IL-6 shRNA blocking cell migration of A549^B[α]P^ and A549^CSE^. Second, IL-6 regulates cigarette smoke lung cancer-promoted osteoclast formation, confirmed by using IL-6 antibody to inhibit A549^B[α]P^ and A549^CSE^ CM-induced osteoclastogenesis. IL-6 is recognized as a critical growth factor in different types of cancer-related osteolytic bone metastasis 52. For instance, IL-6 has been demonstrated to regulate osteolytic bone invasion in human oral cancer cells 53. IL-6 regulates paracrine-autocrine signaling between cancer cells and bone cells, thereby promoting breast cancer-related osteolytic bone metastasis 30, 54. Targeting IL-6 suppress prostate cancer progression in bone 55. Finally, IL-6 controls bone resorption activity and regulates lung cancer-related osteolytic bone metastasis 56. Taken together, these reports and our findings suggest that pharmacological and genetic blockade of IL-6 is a novel avenue for treating smoke-induced lung cancer-related osteolytic bone metastasis.

The PI3K and Akt signaling pathways, activating NF-κB, have close ties to cell proliferation and differentiation, making them crucial contributors to the cell signal transduction network 57. Here, the acute phase response signaling, containing PI3K, Akt and NF-κB, is a top candidate signaling pathway in the GSE31210 database using IPA. Lung cancer cells applied with PI3K, Akt, and NF-κB pharmacological inhibitors all suppress the cigarette smoke-induced promotion of IL-6 generation and cell migration. In addition, the genetic siRNAs have similar effects. Cigarette smoke stimulation facilitates p85, Akt, and p65 phosphorylation. The PI3K and Akt inhibitors abolished the cigarette smoke-mediated luciferase activity of NF-κB, indicating that the PI3K, Akt-activating NF-κB mediate cigarette smoke-induced promotion of IL-6 synthesis and cell motility in human lung cancer. There is an increasing body of report suggesting that the PI3K, Akt, and NF-κB pathways play essential roles in the cancer metastasis. First, the BDNF/TrkB axis facilitates integrin-dependent chondrosarcoma migration via PI3K, Akt, and NF-κB signaling 58. Secondly, the adipokine leptin promotes cell motility of prostate cancer cells through the PI3K/Akt/NF-κB pathway 59. Finally, CCL5 regulates PI3K, Akt, and NF-κB cascades, facilitating lung cancer migration 2. Our previous publication documented that EGFR and c-MET are upstream molecules of the PI3K/Akt pathway in B[α]P and CSE-regulated lung cancer chemoresistance 21. We therefore suggest that EGFR and c-MET are upstream molecules involved in B[α]P- and CSE-induced activation of the PI3K/Akt signaling pathway, thereby promoting IL-6 generation and cell migration.

**Figure 8:**
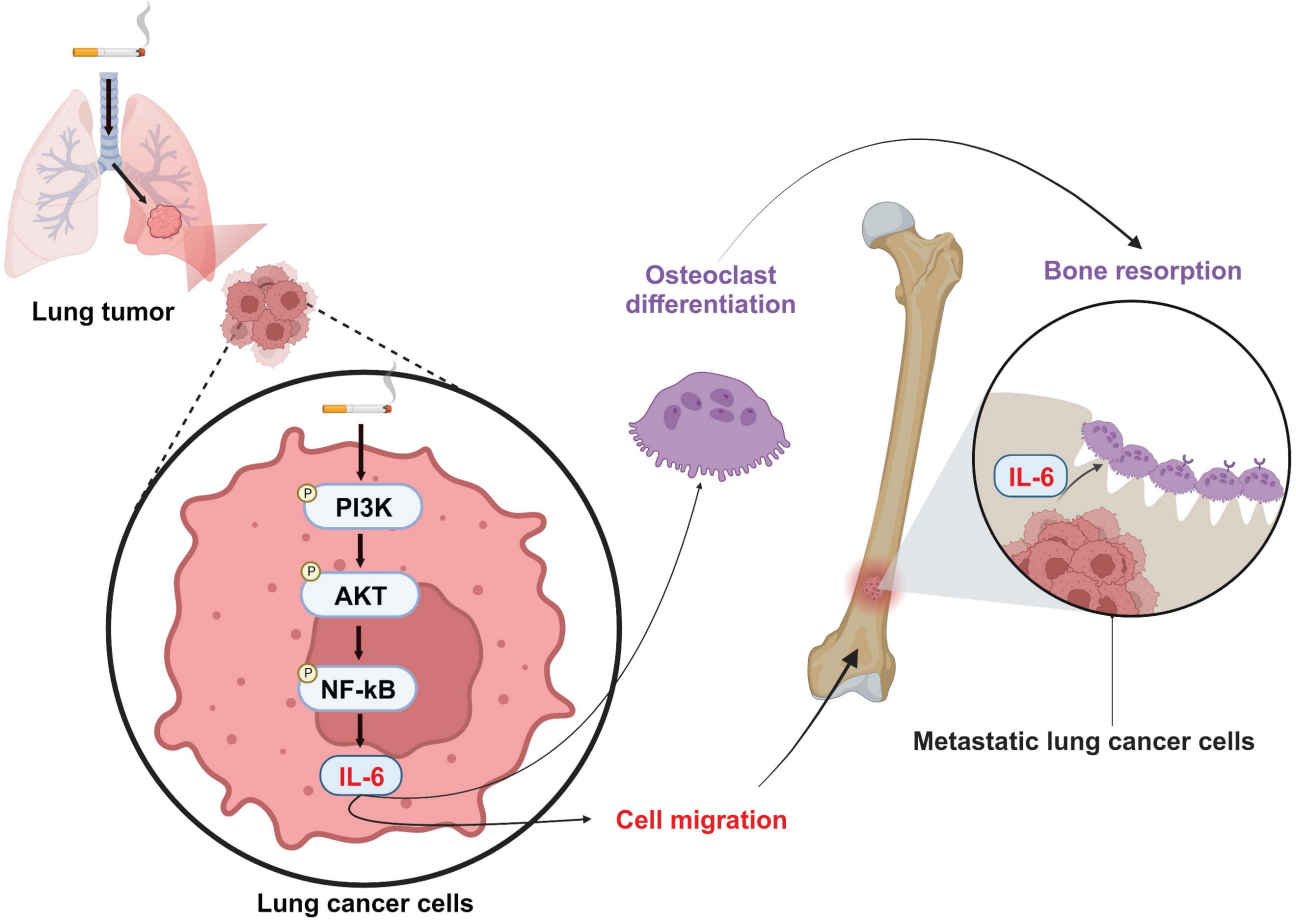
** Schematic diagram summarizing the mechanisms by which cigarette smoke promotes IL-6-dependnet lung cancer osteolytic bone metastasis.** Cigarette smoke promotes IL-6-dependent lung cancer migration through PI3K, Akt and NF-κB signaling pathways. The IL-6 secreted by metastatic lung cancers enhances osteoclastogenesis, leading to osteolytic bone metastasis.

In conclusion, our results disclose that cigarette smoking promotes IL-6-dependent lung cancer migration through PI3K, Akt and NF-κB signaling pathways. The IL-6 secreted by metastatic lung cancers enhances osteoclastogenesis, leading to osteolytic bone metastasis (Fig. 8). Thus, inhibiting IL-6 may be a valuable therapeutic strategy in managing osteolytic bone metastasis in lung cancer patients who smoke.

## Supplementary Material

Supplementary figures.

## References

[1] Thandra KC, Barsouk A, Saginala K, Aluru JS, Barsouk A (2021). Epidemiology of lung cancer. Contemp Oncol (Pozn).

[2] Huang CY, Fong YC, Lee CY, Chen MY, Tsai HC, Hsu HC (2009). CCL5 increases lung cancer migration via PI3K, Akt and NF-kappaB pathways. Biochem Pharmacol.

[3] Daylan AEC, Miao E, Tang K, Chiu G, Cheng H (2023). Lung Cancer in Never Smokers: Delving into Epidemiology, Genomic and Immune Landscape, Prognosis, Treatment, and Screening. Lung.

[4] Spyratos D, Zarogoulidis P, Porpodis K, Tsakiridis K, Machairiotis N, Katsikogiannis N (2013). Occupational exposure and lung cancer. J Thorac Dis.

[5] Kelley DE, Boynton MH, Noar SM, Morgan JC, Mendel JR, Ribisl KM (2018). Effective Message Elements for Disclosures About Chemicals in Cigarette Smoke. Nicotine Tob Res.

[6] Stephens EKH, Guayco Sigcha J, Lopez-Loo K, Yang IA, Marshall HM, Fong KM (2023). Biomarkers of lung cancer for screening and in never-smokers-a narrative review. Transl Lung Cancer Res.

[7] Daniel M, Keefe FJ, Lyna P, Peterson B, Garst J, Kelley M (2009). Persistent smoking after a diagnosis of lung cancer is associated with higher reported pain levels. J Pain.

[8] Fares J, Fares MY, Khachfe HH, Salhab HA, Fares Y (2020). Molecular principles of metastasis: a hallmark of cancer revisited. Signal Transduct Target Ther.

[9] Seyfried TN, Huysentruyt LC (2013). On the origin of cancer metastasis. Crit Rev Oncog.

[10] Al Husaini H, Wheatley-Price P, Clemons M, Shepherd FA (2009). Prevention and management of bone metastases in lung cancer: a review. J Thorac Oncol.

[11] Zhang L, Gong Z (2017). Clinical Characteristics and Prognostic Factors in Bone Metastases from Lung Cancer. Med Sci Monit.

[12] Schmid-Alliana A, Schmid-Antomarchi H, Al-Sahlanee R, Lagadec P, Scimeca JC, Verron E (2018). Understanding the Progression of Bone Metastases to Identify Novel Therapeutic Targets. International journal of molecular sciences.

[13] Kingsley LA, Fournier PG, Chirgwin JM, Guise TA (2007). Molecular biology of bone metastasis. Mol Cancer Ther.

[14] Lyu H, Jundi B, Xu C, Tedeschi SK, Yoshida K, Zhao S (2019). Comparison of Denosumab and Bisphosphonates in Patients With Osteoporosis: A Meta-Analysis of Randomized Controlled Trials. J Clin Endocrinol Metab.

[15] Casas A, Llombart A, Martin M (2013). Denosumab for the treatment of bone metastases in advanced breast cancer. Breast.

[16] Benjamin B, Benjamin MA, Swe M, Sugathan S (2016). Review on the comparison of effectiveness between denosumab and bisphosphonates in post-menopausal osteoporosis. Osteoporos Sarcopenia.

[17] Tammemagi MC, Berg CD, Riley TL, Cunningham CR, Taylor KL (2014). Impact of lung cancer screening results on smoking cessation. J Natl Cancer Inst.

[18] Wu ZJ, Zhao P, Liu B, Yuan ZC (2016). Effect of Cigarette Smoking on Risk of Hip Fracture in Men: A Meta-Analysis of 14 Prospective Cohort Studies. PloS one.

[19] Law MR, Hackshaw AK (1997). A meta-analysis of cigarette smoking, bone mineral density and risk of hip fracture: recognition of a major effect. BMJ.

[20] Jiang YJ, Chao CC, Chang AC, Chen PC, Cheng FJ, Liu JF (2022). Cigarette smoke-promoted increases in osteopontin expression attract mesenchymal stem cell recruitment and facilitate lung cancer metastasis. J Adv Res.

[21] Tu CY, Cheng FJ, Chen CM, Wang SL, Hsiao YC, Chen CH (2018). Cigarette smoke enhances oncogene addiction to c-MET and desensitizes EGFR-expressing non-small cell lung cancer to EGFR TKIs. Mol Oncol.

[22] Liu C-L, Ho T-L, Fang S-Y, Guo J-H, Wu C-Y, Fong Y-C (2023). Ugonin L inhibits osteoclast formation and promotes osteoclast apoptosis by inhibiting the MAPK and NF-κB pathways. Biomedicine & Pharmacotherapy.

[23] Lee H-P, Wu Y-C, Chen B-C, Liu S-C, Li T-M, Huang W-C (2020). Soya-cerebroside reduces interleukin production in human rheumatoid arthritis synovial fibroblasts by inhibiting the ERK, NF-κB and AP-1 signalling pathways. Food Agr Immunol.

[24] Liu S-C, Tsai C-H, Wu T-Y, Tsai C-H, Tsai F-J, Chung J-G (2019). Soya-cerebroside reduces IL-1β-induced MMP-1 production in chondrocytes and inhibits cartilage degradation: implications for the treatment of osteoarthritis. Food Agr Immunol.

[25] Liu SC, Chiu CP, Tsai CH, Hung CY, Li TM, Wu YC (2017). Soya-cerebroside, an extract of Cordyceps militaris, suppresses monocyte migration and prevents cartilage degradation in inflammatory animal models. Sci Rep-Uk.

[26] Lee HP, Wang SW, Wu YC, Tsai CH, Tsai FJ, Chung JG (2019). Glucocerebroside reduces endothelial progenitor cell-induced angiogenesis. Food Agr Immunol.

[27] Su C-H, Lin C-Y, Tsai C-H, Lee H-P, Lo L-C, Huang W-C (2021). Betulin suppresses TNF-α and IL-1β production in osteoarthritis synovial fibroblasts by inhibiting the MEK/ERK/NF-κB pathway. Journal of Functional Foods.

[28] Lee HP, Chen PC, Wang SW, Fong YC, Tsai CH, Tsai FJ (2019). Plumbagin suppresses endothelial progenitor cell-related angiogenesis in vitro and in vivo. Journal of Functional Foods.

[29] Altin A, Zi̇hni̇ Korkmaz M, Atak M, Mercantepe T, Yilmaz H (2023). Celastrol restricts experimental periodontitis related alveolar bone loss by suppressing inflammatory cytokine response. BioMedicine.

[30] Chen WC, Chang AC, Tsai HC, Liu PI, Huang CL, Guo JH (2023). Bone sialoprotein promotes lung cancer osteolytic bone metastasis via MMP14-dependent mechanisms. Biochem Pharmacol.

[31] Liu S-C, Hsieh H-L, Tsai C-H, Fong Y-C, Ko C-Y, Wu H-C (2022). CCN2 Facilitates IL-17 Production and Osteoclastogenesis in Human Osteoarthritis Synovial Fibroblasts by Inhibiting miR-655 Expression. Journal of Bone and Mineral Research.

[32] Wu TJ, Chang SL, Lin CY, Lai CY, He XY, Tsai CH (2022). IL-17 Facilitates VCAM-1 Production and Monocyte Adhesion in Osteoarthritis Synovial Fibroblasts by Suppressing miR-5701 Synthesis. International journal of molecular sciences.

[33] Wu CY, Ghule SS, Liaw CC, Achudhan D, Fang SY, Liu PI (2023). Ugonin P inhibits lung cancer motility by suppressing DPP-4 expression via promoting the synthesis of miR-130b-5p. Biomed Pharmacother.

[34] Macaluso M, Paggi MG, Giordano A (2003). Genetic and epigenetic alterations as hallmarks of the intricate road to cancer. Oncogene.

[35] Wu Y-y, Li X-f, Wu S, Niu X-n, Yin S-q, Huang C (2022). Role of the S100 protein family in rheumatoid arthritis. Arthritis Research & Therapy.

[36] Shupp AB, Kolb AD, Mukhopadhyay D, Bussard KM (2018). Cancer Metastases to Bone: Concepts, Mechanisms, and Interactions with Bone Osteoblasts. Cancers.

[37] Thielen A, Klus H, Muller L (2008). Tobacco smoke: unraveling a controversial subject. Exp Toxicol Pathol.

[38] Talhout R, Schulz T, Florek E, van Benthem J, Wester P, Opperhuizen A (2011). Hazardous compounds in tobacco smoke. Int J Environ Res Public Health.

[39] Chang J, Wang Q, Dong X, Luo T, Liu Z, Xu D (2022). The influencing factors of health hazards of benzo[a]pyrene in cigarette mainstream smoke: The example of one brand in Beijing. Tob Induc Dis.

[40] Teitelbaum SL (2000). Bone resorption by osteoclasts. Science.

[41] Eriksen EF (2010). Cellular mechanisms of bone remodeling. Rev Endocr Metab Disord.

[42] Hollenbach KA, Barrett-Connor E, Edelstein SL, Holbrook T (1993). Cigarette smoking and bone mineral density in older men and women. Am J Public Health.

[43] Egger P, Duggleby S, Hobbs R, Fall C, Cooper C (1996). Cigarette smoking and bone mineral density in the elderly. J Epidemiol Community Health.

[44] Sozen T, Ozisik L, Basaran NC (2017). An overview and management of osteoporosis. Eur J Rheumatol.

[45] Garbers C, Aparicio-Siegmund S, Rose-John S (2015). The IL-6/gp130/STAT3 signaling axis: recent advances towards specific inhibition. Curr Opin Immunol.

[46] Soler MF, Abaurrea A, Azcoaga P, Araujo AM, Caffarel MM (2023). New perspectives in cancer immunotherapy: targeting IL-6 cytokine family. J Immunother Cancer.

[47] Metcalfe RD, Putoczki TL, Griffin MDW (2020). Structural Understanding of Interleukin 6 Family Cytokine Signaling and Targeted Therapies: Focus on Interleukin 11. Front Immunol.

[48] Rosell R, Bertran-Alamillo J, Molina MA, Taron M (2009). IL-6/gp130/STAT3 signaling axis in cancer and the presence of in-frame gp130 somatic deletions in inflammatory hepatocellular tumors. Future Oncol.

[49] Dawson RE, Jenkins BJ, Saad MI (2021). IL-6 family cytokines in respiratory health and disease. Cytokine.

[50] Manolagas SC (1995). Role of cytokines in bone resorption. Bone.

[51] Yoneda T, Nakai M, Moriyama K, Scott L, Ida N, Kunitomo T (1993). Neutralizing antibodies to human interleukin 6 reverse hypercalcemia associated with a human squamous carcinoma. Cancer Res.

[52] Tawara K, Oxford JT, Jorcyk CL (2011). Clinical significance of interleukin (IL)-6 in cancer metastasis to bone: potential of anti-IL-6 therapies. Cancer Manag Res.

[53] Tang CH, Chuang JY, Fong YC, Maa MC, Way TD, Hung CH (2008). Bone-derived SDF-1 stimulates IL-6 release via CXCR4, ERK and NF-kappaB pathways and promotes osteoclastogenesis in human oral cancer cells. Carcinogenesis.

[54] Zheng Y, Chow SO, Boernert K, Basel D, Mikuscheva A, Kim S (2014). Direct crosstalk between cancer and osteoblast lineage cells fuels metastatic growth in bone via auto-amplification of IL-6 and RANKL signaling pathways. J Bone Miner Res.

[55] Zheng Y, Basel D, Chow SO, Fong-Yee C, Kim S, Buttgereit F (2014). Targeting IL-6 and RANKL signaling inhibits prostate cancer growth in bone. Clin Exp Metastasis.

[56] Shih LY, Shih HN, Chen TH (2004). Bone resorption activity of osteolytic metastatic lung and breast cancers. J Orthop Res.

[57] Liu S, Ma H, Zhang H, Deng C, Xin P (2021). Recent advances on signaling pathways and their inhibitors in rheumatoid arthritis. Clin Immunol.

[58] Lin CY, Chen HJ, Li TM, Fong YC, Liu SC, Chen PC (2013). beta5 integrin up-regulation in brain-derived neurotrophic factor promotes cell motility in human chondrosarcoma. PloS one.

[59] Huang CY, Yu HS, Lai TY, Yeh YL, Su CC, Hsu HH (2011). Leptin increases motility and integrin up-regulation in human prostate cancer cells. J Cell Physiol.

