# Supplementary material for: Cigarette smoke promotes IL-6-dependent lung cancer migration and osteolytic bone metastasis
Source: Int J Biol Sci. 2024 Jun 3;20(9):3257–68. doi: 10.7150/ijbs.94339 (PMC11234207; doi:10.7150/ijbs.94339)
Supplement: Supplementary file 1 — Supplementary figures. [file ijbsv20p3257s1.pdf]

Supplementary data

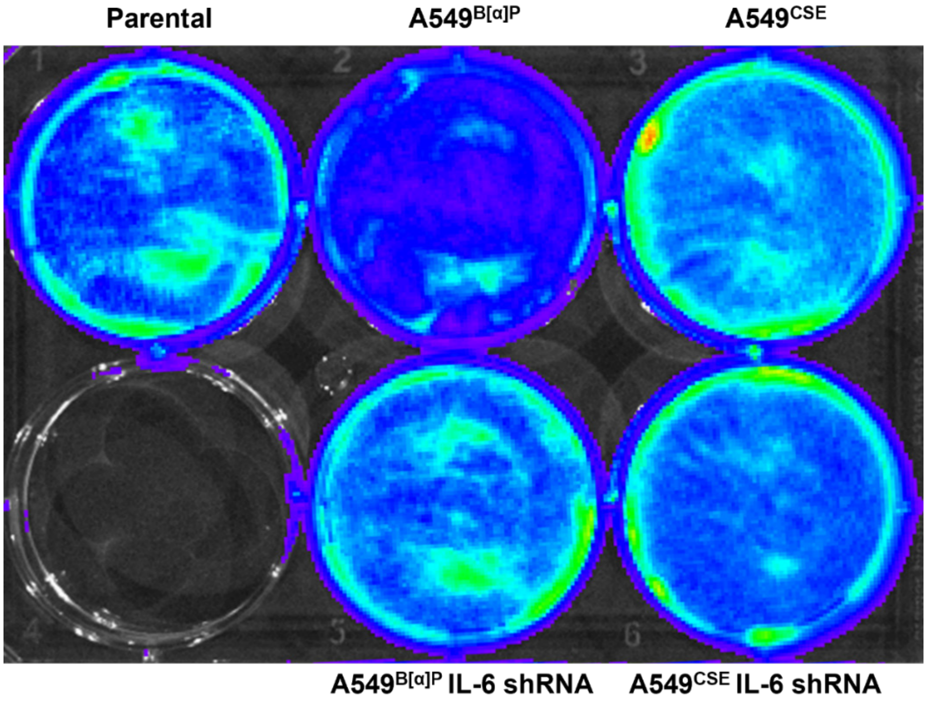

Supplementary Fig. S1. The luciferase activity in indicated cells was examined by luciferase assay.

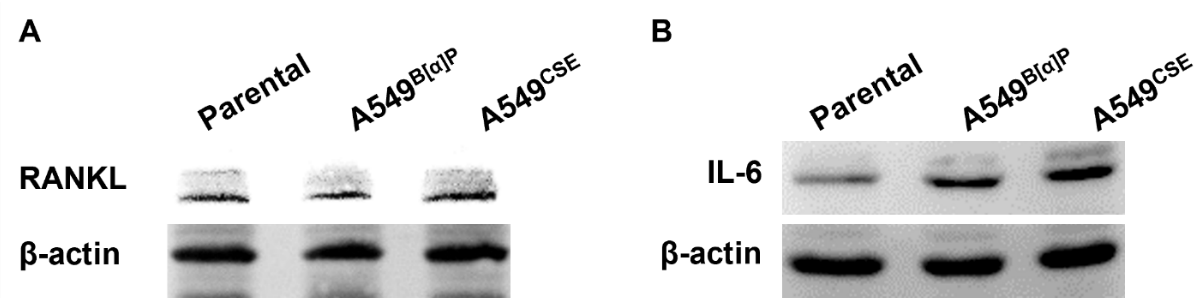

Supplementary Fig. S2. The RANKL and IL-6 expression in indicated lung cancer cells was examined by Western blot.

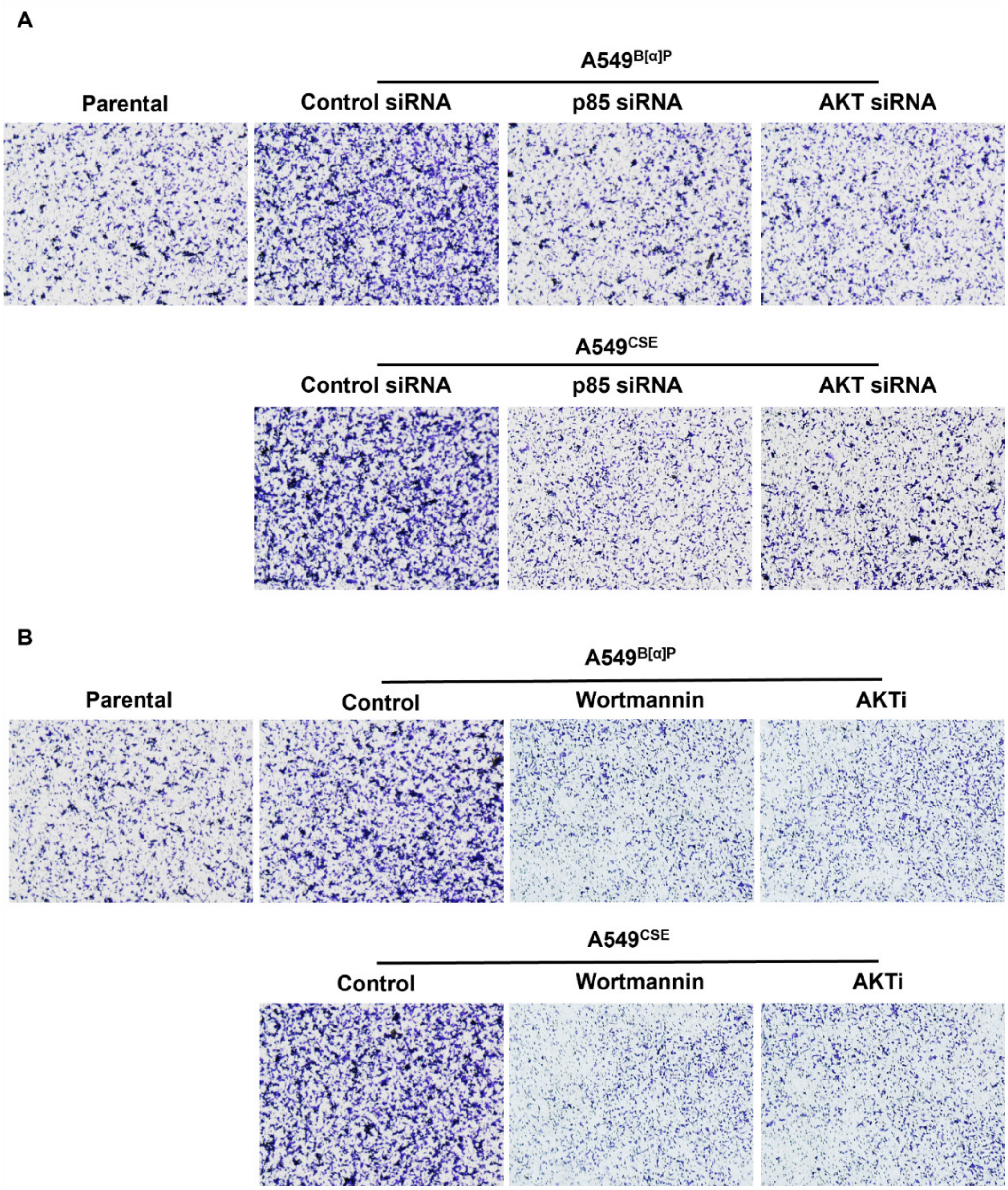

Supplementary Fig. S3. The original images of cell migration assay in Figure 3.

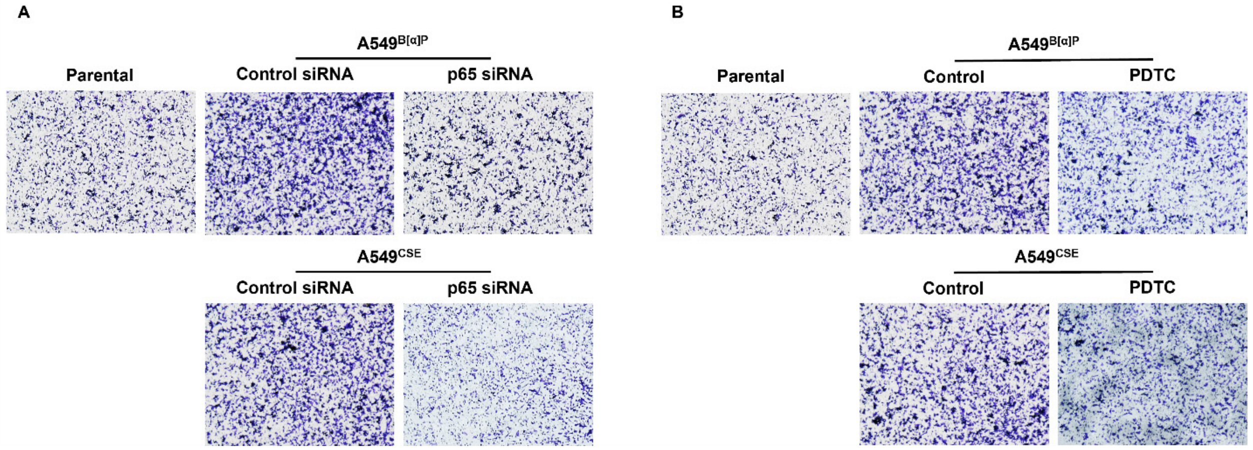

Supplementary Fig. S4. The original images of cell migration assay in Figure 4.

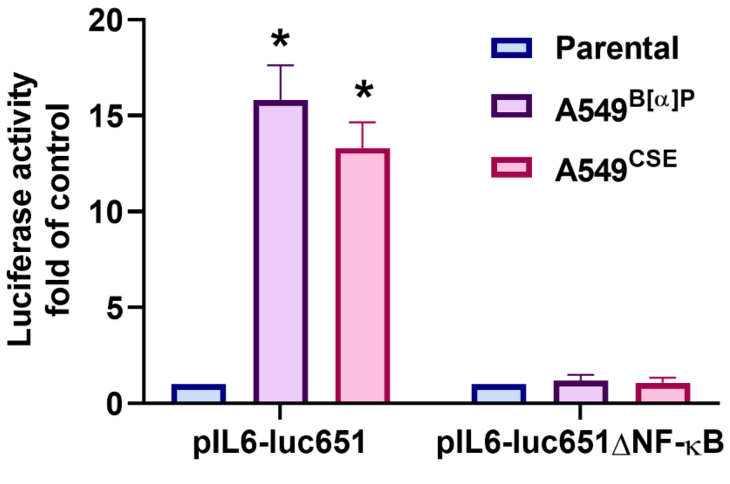

Supplementary Fig. S5. The IL-6 luciferase activity in indicated cells was examined by luciferase activity.

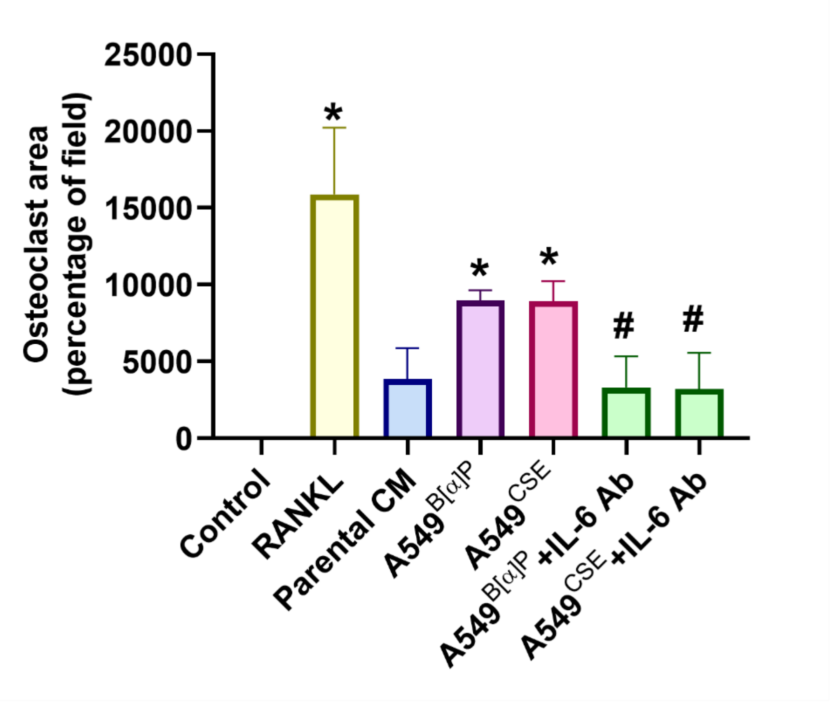

Supplementary Fig. S6. Human primary monocytes were treated with lung cancer CM with or without IL-6 antibody (1 μg/ml) for 7 days, the mature osteoclast was determined and quantified by TRAP staining. *$p < 0.05$ versus the parental group; #$p < 0.05$ versus the A549$^{B[\alpha]P}$ or A549^{CSE} CM group.

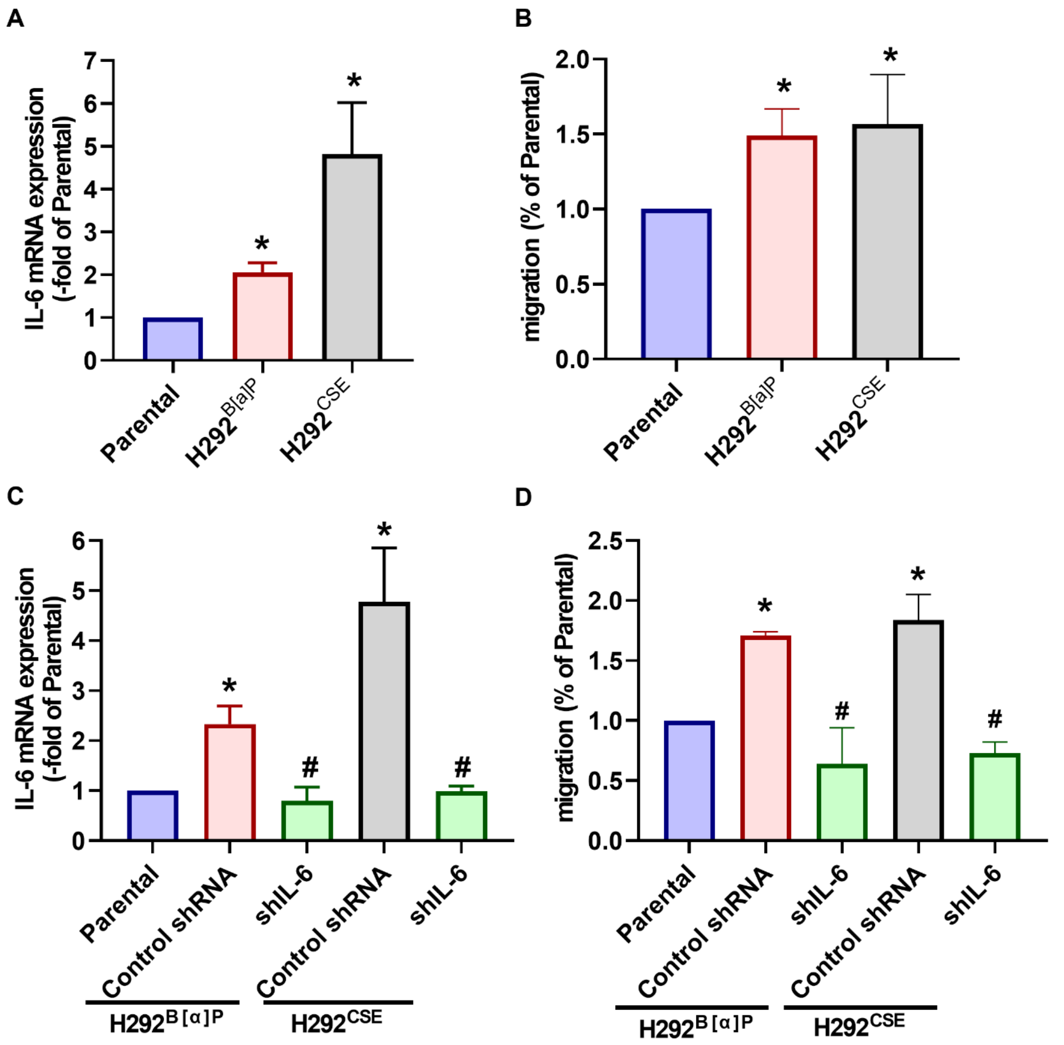

Supplementary Fig. S7. (A&B) The IL-6 mRNA level and cell migration ability in indicated cells was examined. (C&D) Lung cancer cells were transfected with IL-6 shRNA, and IL-6 expression and cell migration were examined by qPCR and migration assay. *$p < 0.05$ versus the parental group; #$p < 0.05$ versus the control-shRNA group.

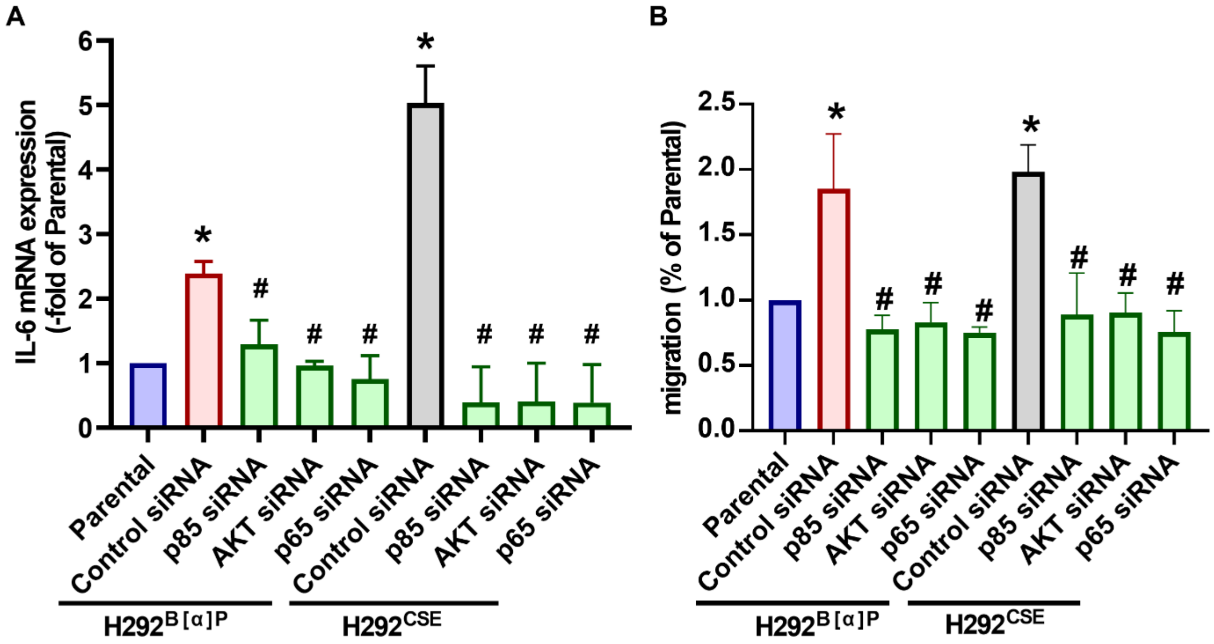

Supplementary Fig. S8. Cells were transfected with p85, Akt or p65 siRNA, and IL-6 expression and cell migration were examined by qPCR and migration assay. *$p < 0.05$ versus the parental group; #$p < 0.05$ versus the control-siRNA group.

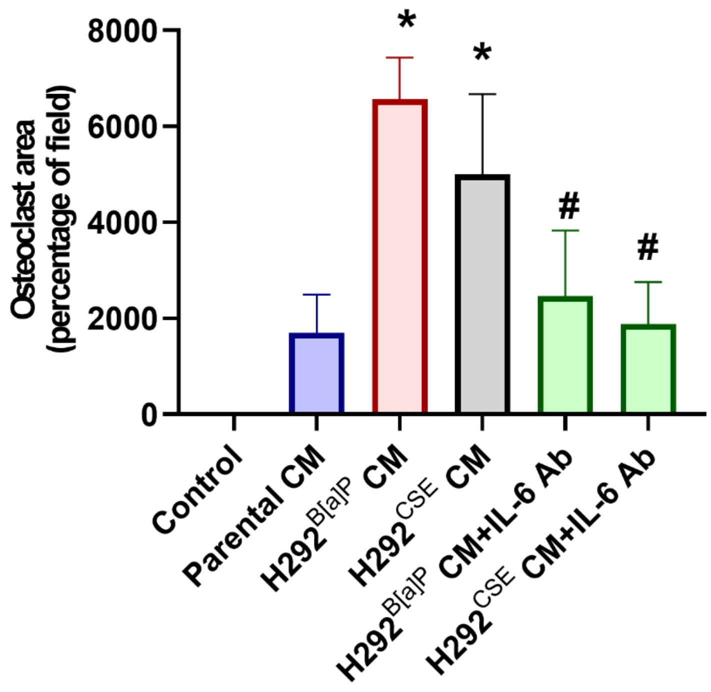

Supplementary Fig. S9. RAW 264.7 cells were treated with lung cancer CM with or without IL-6 antibody (1 μg/ml) for 7 days, the mature osteoclast was determined and quantified by TRAP staining. *p < 0.05 versus the parental group; #p < 0.05 versus the H292$^{B[\alpha]P}$ or H292^{CSE} CM group.
